# The Low Velocity Impact Response of Foam Core Sandwich Panels with a Shape Memory Alloy Hybrid Face-Sheet

**DOI:** 10.3390/ma11112076

**Published:** 2018-10-24

**Authors:** Hao Li, Zhenqing Wang, Zhengwei Yu, Min Sun, Yanfei Liu

**Affiliations:** 1 College of Aerospace and Civil Engineering, Harbin Engineering University, Harbin 150001, China; lihao0202@hrbeu.edu.cn (H.L.); yzw0514@hrbeu.edu.cn (Z.Y.); sunmin@hrbeu.edu.cn (M.S.); 2 State Key Laboratory of Tribology, Tsinghua University, Beijing 100084, China; yanfeiliu@hrbeu.edu.cn

**Keywords:** sandwich panel, shape memory alloy, hybrid face-sheet, low velocity impact

## Abstract

Most foam core sandwich panels are sensitive to the impact load because of the poor toughness of thin composite face-sheets and the low strength of foam core. Superelastic shape memory alloy (SMA) wires have been applied to enhance the impact damage resistance of composite laminates in recent decades. To improve the impact damage resistance of foam core sandwich panels and to protect the foam core, SMA wires were incorporated into the face-sheets of foam core sandwich panels in this work. Eight new types of SMA hybrid sandwich panels were designed, and low-velocity impact tests were carried out at an impact energy of 35 J. The damage morphology of the impacted sandwich panels was identified by visual inspection and scanning electron microscope technology. Results indicate that the impact damage resistance of the SMA hybrid sandwich panels is enhanced. The damage area in the hybrid sandwich panels is greatly reduced and a decrease of 85.63% can be reached in the bottom face-sheet. The maximum contact force has an improvement of 28.15% when the two layers of SMA wires are incorporated into the bottom face-sheet.

## 1. Introduction

Due to the excellent mechanical properties in terms of high strength and stiffness to weight ratios, good corrosion resistance and ease of fabricating and repair, composite sandwich structures have been widely applied in many fields such as aerospace, the shipbuilding industry and wind turbine blades in recent decades [1,2,3,4]. However, most sandwich panels are inherently sensitive to localized damage caused by transverse loads, which is attributed to the poor toughness of thin laminated face-sheets and low strength of core materials. Low velocity impact as one type of common localized load can damage sandwich panels. The presence of different damage modes (matrix cracking, fiber breaking, delamination, skin-core debonding, core crushing, etc.) results in a significant decrease in the mechanical performance of sandwich panels [5,6,7,8,9,10]. Therefore, investigation on the impact response of sandwich panels has been an important concern for security and full application of the sandwich structures.

Comprehensive research has been done on the mechanical response of sandwich panels subjected to low velocity impact. The impact damage mechanisms of sandwich panels made of different composite skins and core materials have been investigated by many researchers [11,12,13,14,15,16]. Zhou et al. [11] conducted impact tests on sandwich structures based on layered cores. A range of Polyetherimide (PEI), crosslinked Polyvinyl chloride (PVC) and linear PVC foams were mixed to form a three-layer core. The work showed that the majority of the panels failed in a core shear, leaving an evident cylindrical hole in the combined core. Graded core structures outperformed their monolithic counterparts, and improved impact resistance could be obtained by arranging the high-density foam core against the front face-sheet. Research on the low-velocity impact behavior of sandwich panels with plain woven carbon fabric laminated skins and polyurethane foam core was conducted by Wang et al. [12]. The influences of the projectile size, incident energy, skin thickness and core thickness on the impact response of sandwich panels were also investigated. The impact parameters (peak load, dissipated energy/incident energy ratio and contact duration) increased with incident energy, while the impact behavior and failure state were independent of the core thickness. The low velocity impact tolerance of sandwich structures consisting of stitched foam core and glass skins was evaluated by Lascoup et al. [15]. The existence of stitches considerably enhanced the impact performance of the sandwich structure. The failure of the internal 3D structure dominated the energy consumption mode, resulting in the absence of delamination in the modified sandwich composite. The low velocity impact behavior of foam-filled 3D integrated core sandwich laminates with hybrid skins was studied by Hosur et al. [16]. Four types of hybrid face sheets were tested, and different impact parameters (peak load, deflection, dissipated energy, etc.) were evaluated.

According to the research on the low velocity impact response of sandwich panels, it is evident that the components of sandwich structures (the material of the face sheet, the core thickness, etc.) play important roles on the impact resistance of sandwich panels. To improve the impact tolerance of sandwich panels, some works have been conducted by researchers. For the composite laminated face sheets, the unidirectional fibers were replaced with hybrid woven fabric; as a consequence, the impact tolerance of sandwich panels was modified with high toughness skin [17]. A possible alternative method to enhance the impact resistance of sandwich panels is to use the shape memory alloy (SMA) hybrid composite laminates as the face sheets. Due to the high recoverable and failure strain, the superelastic SMA has been incorporated into the fiber-reinforced composite laminates, and the low velocity impact response of the SMA hybrid composite laminates has been investigated by many researchers [18,19,20,21,22,23]. The work of Pappadà et al. [21] showed that the SMA fibers had a positive influence on the impact resistance of glass reinforced laminates. Aurrekoetxea et al. [22] studied the low-velocity impact response of carbon fiber reinforced composites embedded with SMA. The results indicated that the impact tolerance of SMA hybrid composites is improved as the load capacity of the structure was increased. Pinto and Meo [23] conducted an experimental study on the low velocity impact behavior of carbon fiber reinforced composites incorporated with SMA wires. Their work showed that the stiffness of the hybrid composites increased and the extent of the intra-laminar damage in the SMA reinforced specimens had a large reduction, even up to 300% for the medium incident energies. Therefore, the low velocity impact resistance of sandwich panels with face sheets made of SMA hybrid composites can be improved due to the enhanced impact tolerance of the SMA hybrid composite structures.

In this paper, to enhance the low velocity impact tolerance of foam core sandwich structures, new several structures with SMA hybrid laminated face-sheets were designed. Compared to the traditional face sheet composed of glass fiber reinforced composite laminates, the influences of eight types of SMA hybrid face sheets on the impact response of the new designed sandwich panels were evaluated. One or two layers of SMA wires were incorporated into the face sheet. The impact responses of the designed sandwich panels were reported in terms of contact force-time, displacement-time and energy-time curves. Moreover, the visual inspection method and scanning electron microscopy (SEM) technology were applied to analyze the damage modes of the impacted sandwich panels.

## 2. Materials and Methods

### 2.1. Materials

**Figure 1:**
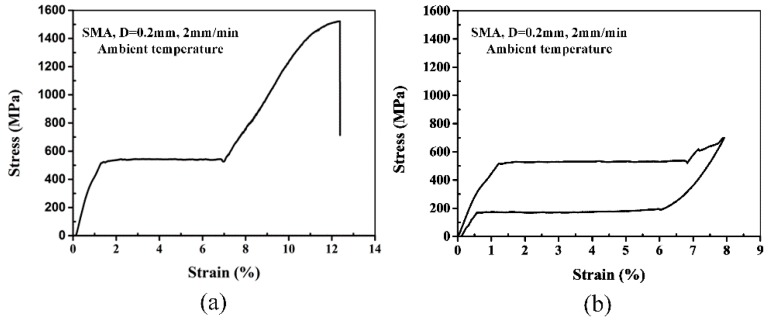
Tensile testing of SMA wires [24]: (**a**) Representative tensile stress-strain curve of SMA wires at breakage and (**b**) superelastic deformation of SAM wires.

In this study, the glass fiber-reinforced composite laminated face sheets with or without SMA wires and a foam core composed the new designed sandwich panels. The polymer applied as the matrix for face sheets was the Vinyl ester resin which can be cured at room temperature mixed with hardening agent and accelerating agent. Methyl ethyl ketone peroxide (MEKP) and Dimethylaniline were used as hardening agent and accelerating agent, respectively. The polymer, hardening agent and accelerating agent were provided by Ashland Inc. (Lexington, KY, USA). The polymer was mixed with the hardening agent and accelerating agent at the weight ratio of 100:1:0.2. The unidirectional glass fiber cloth with a surface density of 200 g/m^2^ and single layer thickness of 0.2 mm was purchased from Tongxiang Mentai Reinforced Composite Material Company (Tongxiang, China). A closed cell PVC foam (DIAB, Laholm, Sweden) with a density of 60 kg/m^3^ and thickness of 6 mm was used as the foam core. The compressive modulus and compressive strength of the foam are 70 MPa and 0.85 MPa, respectively. Moreover, the applied foam core has resin diversion trenches of 20 mm × 20 mm on the surface, which benefits the immersion of resin into the foam during the manufacturing process. The superelastic 55.8 wt.% Ni balance Ti wires applied in this work were the same to our previous research [24], purchased from PeierTech (Jiangyin, China). The diameter of SMA wires is 0.2 mm and the transformation temperatures are *M*_s_ = −17.6 °C, *M*_f_ = −38.2 °C, *A*_s_ = −5.7 °C, *A*_f_ = 11.2 °C. Tensile stress-strain curves of SMA wires are shown in Figure 1. Figure 1a represents the typical tensile stress-strain curve of SMA wires at breakage, and Figure 1b shows the superelastic deformation of SMA wires. In this work, since the transformation temperature *A*_f_ is 11.2 °C, the SMA wires are in the austenitic phase at room temperature. When the load imposed on SMA wires exceeds the critical stress (530.8 MPa in this paper), the austenite phase starts to transform to the martensite phase. Then, the stress induced in the SMA wires is nearly constant over a large strain, creating a plateau region. Finally, when the load is removed, the martensite phase reverts back to the austenite phase, and a large strain (7.9% in this work) is recovered. The main mechanical properties of SMA wires are reported in Table 1.

### 2.2. Sample Manufacturing

Sandwich panels with nine types of composite skins were fabricated by the vacuum-assisted resin injection (VARI) process [25]. The interface adhesion between SAM wires and matrix plays an important role in the mechanical performance of SMA-reinforced composite laminates. Extensive studies have been reported on the improvement of SMA/matrix interface adhesive properties [26,27,28]. In this work, a mechanical treatment was employed to modify the surface properties of SMA wires. The SMA wires were firstly cleaned with acetone for the presence of impurities on the wire surface and were then polished with 180 and 400 grit papers to remove the outside oxide layer. Lastly, the treated SMA wires were embedded into the face sheet with eight modes. The stacking sequences of the sandwich panels investigated are summarized in Table 2. Ply mode I represents the configuration of the reference sandwich panel without SMA wires, ply mode II to ply mode V display the panels with one layer of SMA wires, and the other modes show the panels with two layers of SMA wires. It is worth noting that the two embedded layers SMA wires were perpendicular to each other for balancing the hybrid sandwich panels. All the SMA wires with a gap of 0.3 mm were aligned along the ply orientation of adjacent layers, which can make the SMA wires compatible with surrounding glass fibers upon curing [29]. The volume fraction of wires was 0.14% for one layer and 0.28% for two layers.

**Figure 2:**
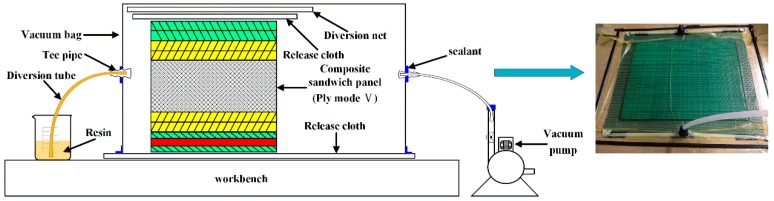
The schematic diagram of the VARI process.

The schematic diagram of the VARI process is showed in Figure 2. During the manufacturing process, a glass plate placed on a table was used as the holder. Firstly, sandwich panels and one layer release cloth on each side of the panel were placed on the holder. Then, one layer of diversion net was covered on the top. Finally, the whole structure was sealed by a vacuum bag and sealant tape. To ensure the resin can flow uniformly, two delivery pipes were fixed at the entrance and exit, respectively. After the infusion of resin, the system was cured at room temperature and a vacuum level of 600 mbar for 20 h. To test the low-velocity impact response of the hybrid sandwich panels, specimens of 100 × 100 × 7.6 mm^3^ were cut from the manufactured panels using a diamond saw blade cutting machine.

### 2.3. Low Velocity Impact Test

**Figure 3:**
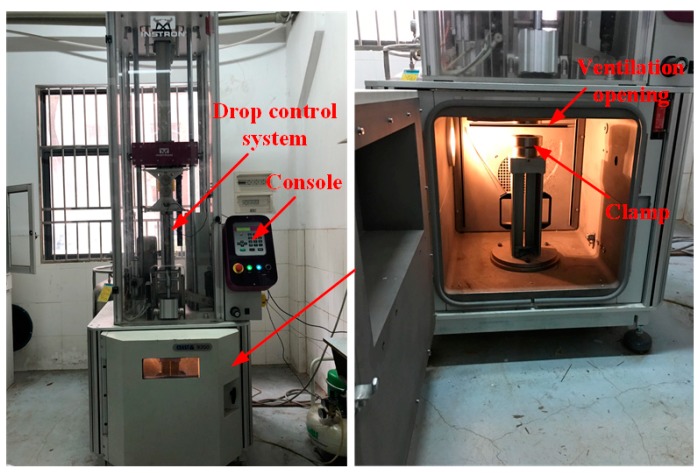
Testing device of the impact tests.

Low velocity impact tests were performed by an Instron Dynatup CEAST 9350 (Instron, Norwood, MA, USA) drop weight impact testing machine at room temperature, as shown in Figure 3. The test system includes three parts: the pneumatic clamping fixture, a drop hammer device and a data acquisition system. The edges of the sandwich samples were firmly fastened between two circular rings with a diameter of 76 mm. The test samples were impacted at the center by a hemispherical projectile according to the American Society for Testing and Materials (ASTM) D5420-2010 standard. The hemispherical impactor with a mass of 3.77 Kg was guided through two smooth columns, which restrained the samples from a second strike. During the test, the contact force was determined by a load cell located just above the impactor head, and the displacement was measured by a laser detector attached on the impact frame. The projectile diameter and impact incident energy are two important impact parameters. In this work, the diameter of the projectile was 16 mm, which is the common size of the impactor. The impact energy level 35 J was selected, resulting in the initial incident velocity 4.31 m/s for the projectile that the sandwich structures usually encounter during their service life. For each type of sandwich panels, at least three samples were tested and the average values were determined.

### 2.4. Damage Morphology Observation

During the impact event, some internal and external damage may occur in the face sheets and foam core, such as matrix cracking, delamination, fiber failure, SMA breakage, skin-core interface debonding and foam crushing. After the impact, the damage modes and extent in the sandwich panels were assessed by visual inspection and sectioning with SEM (Hitachi S-4300, Tokyo, Japan) methods. The damage on the specimens’ front and rear surface can be evaluated by visual inspection. SEM technology was used to observe the micro-damage morphology in the cross sections of the skin. The impacted specimens were cut through at the centers of the damage areas; then, images were taken by SEM at different resolutions to study the damage in detail.

## 3. Results and Discussions

### 3.1. Damage Morphology of the Impacted Foam Core Sandwich Panels

**Figure 4:**
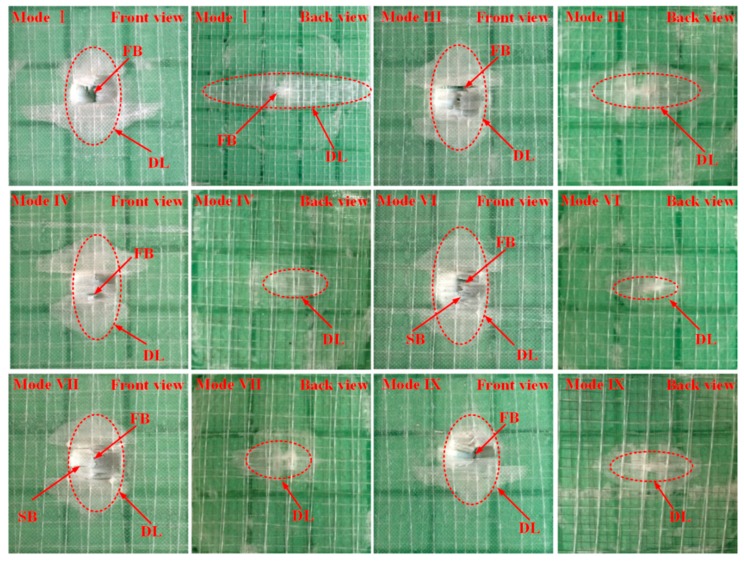
Representative damaged sandwich panels in front and back views. Notes: except the figure back view of Mode I, other figures present the center 60 × 60 mm^2^ region of impacted sandwich panels. DL indicates delamination, FB represents fiber breaking and SB stands for SMA wire breaking.

**Figure 5:**
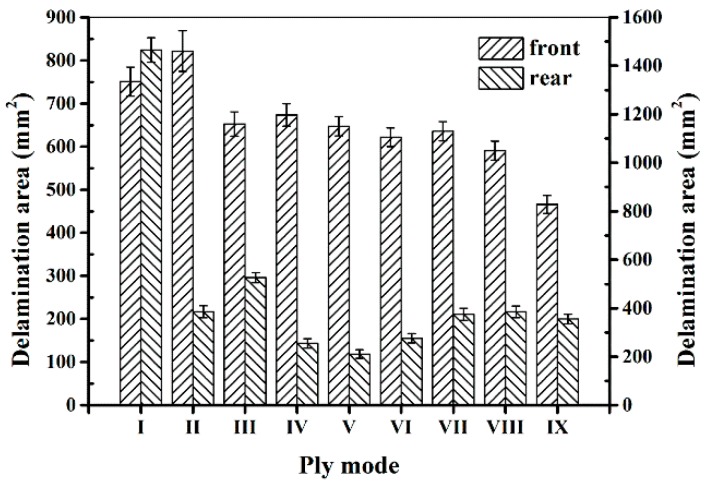
Delamination areas in the front and rear face-sheets of different sandwich panels.

After the impact tests, the damage modes and extent on the front and rear surface of sandwich panels can be distinguished by visual inspection. Typical damage morphology of different sandwich panel modes is recorded in Figure 4. It can be seen that there is a serious damage region on the front surface for all panel modes due to the penetration of the front face-sheet. In general, there is delamination and fiber breaking on the front surface for all modes, and the damage regions do not not have large differences between them. On the front surfaces of mode VI and VII, SMA breaking also occurs at the impact region beneath the impactor. It should be noted that the indentation diameter is a little larger than 16 mm, and the indentation depth of mode I is obviously greater than that of other SMA hybrid modes. For the rear surface, the damage is relatively weak because the bottom face-sheet has not been penetrated. The main damage type is delamination. For mode I, fiber breaking also appears at the center of the rear surface. Generally, the impact damage area has a peanut shape in the front face-sheet and an elliptical shape in the bottom face-sheet, with the long edge orienting the fiber direction of the lower ply where adjacent plies has different fiber directions. Similar damage behavior has been indicated in research on low-velocity impact of fiber-reinforced composite laminates [30,31]. The damage size of different sandwich panels is reported by the damage area, determined from the figures by visual inspection and plotted in Figure 5. From Figure 5, it can be seen that the delamination areas in the front face-sheets of SMA hybrid sandwich panels are smaller than the mode I baseline sandwich panel. Specifically, the damage area of mode IX has a decrease of 37.93% compared to mode I. For the delamination areas in the bottom face-sheets, there is a significant decrease for all SMA hybrid sandwich panels and the decrease even reaches 85.63% for mode V. For SMA hybrid sandwich panels, the sandwich panels embedded with two layers of SMA wires have a smaller damage area relative to the panels with one layer of SMA wires in the front face-sheets, and there is no clear trend for the damage area of bottom face-sheets. The impact damage resistance of hybrid face-sheets is improved due to the presence of SMA wires, which decreases the tendency of the panels to consume impact energy through delamination. Specially, when SMA wires are incorporated into the bottom face-sheet, the mechanical performance of SMA wires can be effectively exerted on the lower part of the sandwich panel sustain tensile stress, resulting in a significant decrease of the delamination area. Similar results have been obtained for the low-velocity impact behavior of SMA hybrid composite laminates in reported work [23,29].

**Figure 6:**
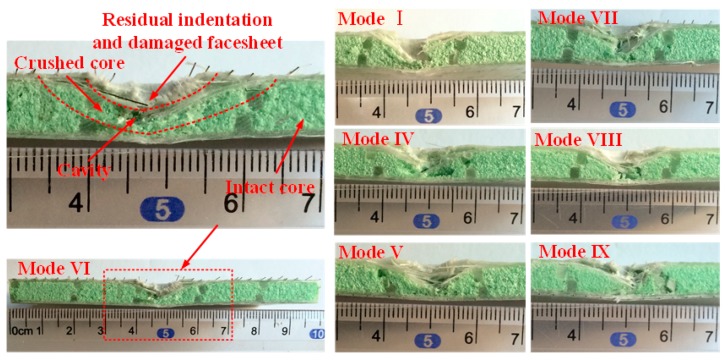
The transverse damage morphology of representative impacted sandwich panels.

**Figure 7:**
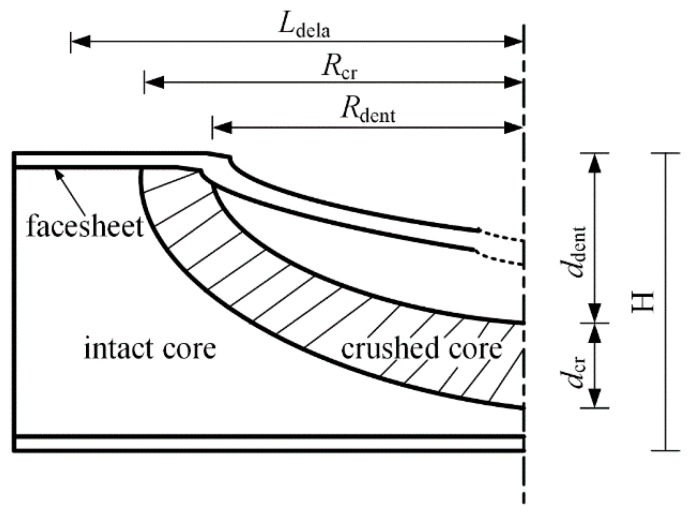
The cross-section schematic diagram throughout the damage region (*L*_dela,_ *R*_cr_, and *R*_dent_ represents the length of the delamination, the radii of the crushed core and the indentation, respectively. *d*_dent_ and *d*_cr_ indicate the depths of indentation and crushed core, respectively. H stands for the thickness of the sandwich panel).

The typical cross-sections of the impacted samples are shown in Figure 6. As reported by Shipsha and Zenkert [32], for foam-core sandwich panels subjected to low-velocity impact, the damage modes mainly include permanent indentation on the front surface, delamination between plies, fiber breaking and a partially crushed core around the impacted region. The schematic diagram of the damaged panel cross-section is shown in Figure 7. For panels investigated in this work, as the projectile has penetrated the front face-sheets, the residual indentation can be clearly seen right beneath the impactor. Local matrix crushing, delamination and fiber breaking can also be identified in the face-sheets. For hybrid sandwich panels with SMA wires embedded in the front face-sheet, the failure of SMA wires also occurs. For the foam core, partially crushed foam core accompanied with a cavity is clearly revealed around the indentation region. Compared to mode I, the extent of the damage in the SMA hybrid sandwich panels is weaker, which can be indicated by the smaller crushed foam core and the shallower indentation depth. With a close observation, it can also be found that the damage extent of the hybrid panels with two layers of SAM wires is weaker than that of the panels with one layer of SMA wires. Specifically, the indentation depth of the mode IX is just about 3 mm, half of the foam core thickness, and the permanent deformation of the foam core around the damage region is weak and concentrated. The impact damage resistance of the SMA hybrid composite skins is improved with higher strength and stiffness relative to the conventional composite laminates, which has been reported in other research [33,34]. Due to the presence of SMA wires, the deflection of the front face-sheet is reduced, and the face-sheet with higher strength can offer a much better protection against the compression of the foam core, thus reducing or preventing the damage of the foam core. With SMA wires embedded into the bottom face-sheet, the impact load can be effectively transferred to the superelastic SMA wires which have high strength and failure strain; thus, the damage induced by the projectile is largely reduced.

**Figure 8:**
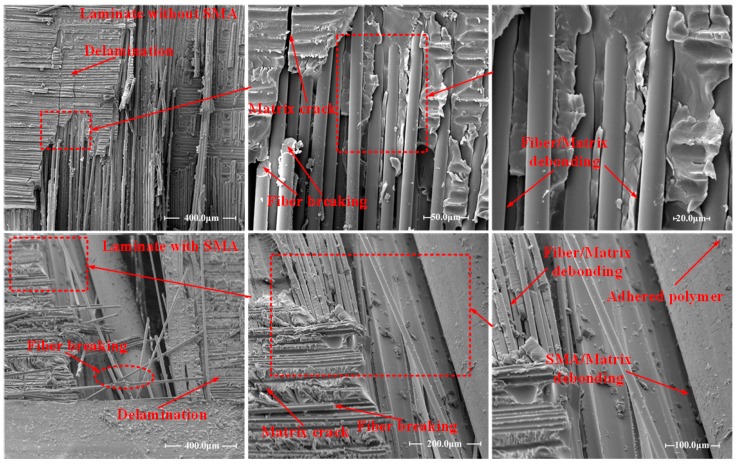
Typical SEM images obtained on the face-sheets.

The macro-damage morphology of the impacted sandwich panels has been shown by visual inspection, and the damage modes have been identified. To further understand the damage mechanism of the sandwich panels subjected to impact loading, SEM technology has been applied to identify the micro-damage morphology of the sandwich structures. As the damage occurring in the foam core mainly includes foam crushing or cracking which can be clearly indicated by visual inspection, the SEM images have only taken on the composite face-sheets around the damage region. The SEM figures obtained are shown in Figure 8. For face-sheets with or without SMA wires, delamination and fiber breaking can be clearly indicated. For the reference face-sheet, micro-matrix cracking and fiber/matrix debonding can be identified in the enlarged images. While for the SMA hybrid face-sheet, the SMA wire/matrix debonding can also be distinguished. As matrix cracks propagate rapidly along the interface between SMA wire and matrix, a large separation region between the SMA wire and the surrounding composite is observed.

During an impact event, different impact responses of the sandwich panels occur in sequence with load increasing. The damage evolution for the conventional sandwich panel without SMA wires can be described as follows. Firstly, since the load imposed on the structure is low, the whole sandwich panel deform elastically. There is almost no damage occurrence in the structure. Secondly, with the deflection of the front face-sheet increasing, the foam core begins to yield and crush due to the low strength and poor initial compression resistance. At this stage, there is still no evident damage induced in the face-sheets. As reported in [12,35], the initial primary damage mode occurring in the sandwich panel is the foam core crushing, which leads to the initial structure stiffness change and may result in a sudden contact force drop. The presence of damage at this stage cannot cause catastrophic failure of the sandwich panel. Thirdly, with the projectile gradually penetrating the front face-sheet, matrix cracking and delamination between plies occur in both face-sheets, fiber breaking is also induced in the front face-sheet. At the same time, the progressive collapse of the foam is induced by the indentation of the front face-sheet (Figure 4). The damage induced in the face-sheets is similar to the fiber-reinforced composite laminate subjected to low-velocity impact and has been indicated in other studies [30,31]. For the face-sheets of sandwich panels, matrix cracks induced by shear or tensile load, extend in plies or propagate along the fiber/matrix interfaces. When matrix cracks reach the interface between plies with different orientation, delamination is caused when the interface cohesive strength is reached. With extensive extension of delamination and matrix cracks, fiber breaking appears when the tensile strength of fibers is exceeded. Fourthly, with much more severe matrix cracks and fiber breaks, the projectile penetrates the front face-sheet and a circular shaped hole is formed. There is also a much larger area of oam crushing under the front face-sheet due to the penetration of the impactor (Figure 6). Lastly, the impactor almost reaches the bottom face-sheet and then rebounds. Compared to the reference sandwich panel, the plastic deformation and failure of SMA wires, and SMA/matrix debonding are induced in the sandwich panels embedded with SMA wires. The SMA/matrix debonding illustrated by the SEM images in Figure 8 occurs when the cohesive interface strength of SMA/matrix is exceeded. After the occurrence of SMA/matrix debonding, matrix cracks can also extend along the SMA/matrix interface. With SMA wires incorporated into the front face-sheet, failure of SMA wires appears when the impactor cuts through the front face-sheet. Although the SMA wires in the bottom face-sheet are not damaged, large plastic deformation is activated. All these impact responses generated with SMA wires are beneficial for the dissipation of the impact energy. The impact damage resistance of SMA hybrid sandwich panels is improved due to the presence of SMA wires.

### 3.2. Impact Responses of Hybrid Sandwich Panels with One Layer of SMA Wires

**Figure 9:**
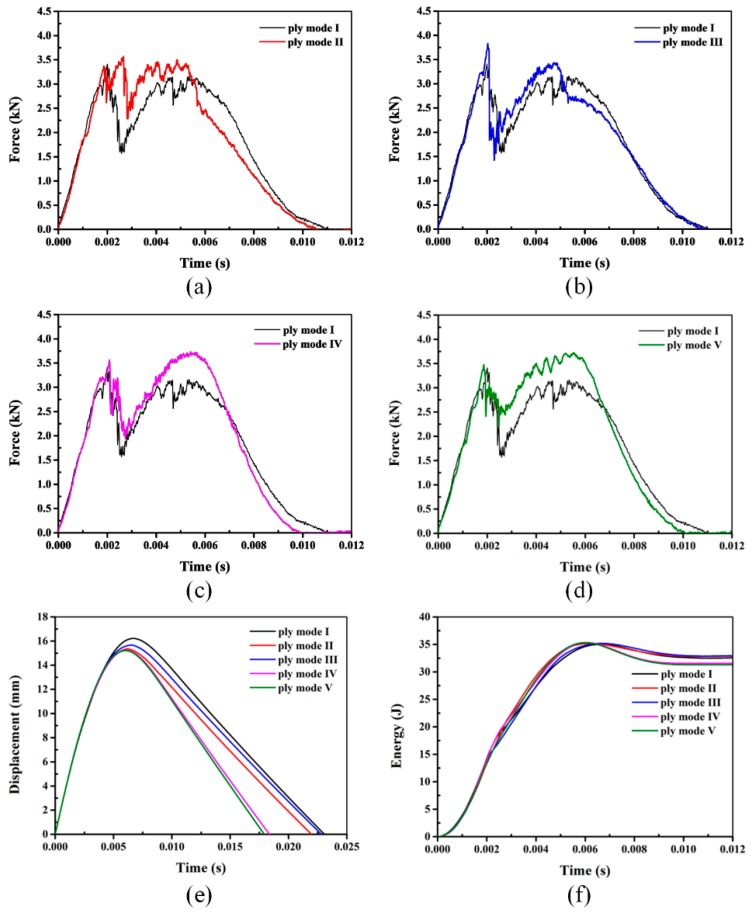
Typical impact responses of hybrid sandwich panels with one layer of SMA wires ((**a–d**) represent the force-time curves of mode I with modes II, mode III, mode IV and mode V, respectively. (**e**,**f**) show the displacement-time and energy-time histories, respectively).

The typical impact responses of hybrid sandwich panels with one layer of SMA wires are shown in Figure 9. Contact force as an important parameter for impact problems can be used to evaluate the impact damage tolerance of sandwich panels for the load bearing capability of the structures assessed by the maximum contact force [11]. Two peak forces are observed in the force-time curves of Figure 9. Combining the damage morphology of sandwich panels, the first peak force clearly indicates the penetration of the front face-sheet and the second reveals the indentation of the impactor into the foam core. Based on the testing data, the maximum forces and displacements together with absorbed energies are summarized in Table 3. The results consistently indicate that the maximum forces of hybrid sandwich panels are larger than the baseline panel. The maximum force of mode III can reach 3.78 kN, which shows an increase of 10.85% compared to the reference panel. The maximum forces of both mode IV and V are comparable to that of mode III, and the contact force after the first peak force is much larger than that of mode III. For mode III, the first peak force as the maximum force indicates that the tolerance of the front face-sheet to support the impact load is improved due to the hybridization of SMA wires. While for both modes IV and V, the SMA wires are incorporated into the bottom face-sheet, and the maximum force is located at the second peak force. Since the embedded SMA wires in the bottom face-sheet sustain tensile load and deform largely, the contact force around the second peak force is much larger than that in mode I. The mechanical properties of the hybrid face-sheets are enhanced due to the superior mechanical performance of SMA wires, which is indicated by Liu et al. [33]. Moreover, the high constant stress of SMA wires in the stress-induced martensite transformation process also makes them support a huge amount of impact loading. Therefore, the load-bearing capability of hybrid sandwich panels is improved with enhanced face-sheets. As higher load-bearing capability of sandwich structures indicates an advantage to support a foreign load, the impact damage tolerance of sandwich panels with SMA hybrid face-sheets is increased.

The typical displacement-time curves of sandwich panels are plotted in Figure 9e. The maximum displacement of different panels is concluded in Table 3. The structure with higher bending stiffness will generate smaller deflection. The maximum displacement can be another useful parameter to assess the impact damage resistance of sandwich panels [17]. The maximum displacement of SMA hybrid sandwich panels is smaller than the conventional sandwich panel without SMA wires. In detail, the maximum displacement of modes II, III, IV and V sandwich panels with SMA hybrid face-sheets has a reduction of 5.78%, 3.63%, 6.77% and 7.02%, respectively, relative to the mode I reference sandwich panel. As revealed by Liu et al [33], the flexural strength of hybrid face-sheet is increased because of the existence of SMA wires. Therefore, the bending resistance of sandwich panels with SMA hybrid face-sheet is improved and the maximum displacement is reduced compared to the baseline sandwich panel. The displacement-time histories also show that the time for the projectile to return the contact position is shorter during the hybrid sandwich panel impact event. Especially, compared with the 23 ms of mode I, the time for both modes IV and V is only about 18 ms. Moreover, the recovery stress generated in SMA wires after unloading is beneficial for the recovery of the hybrid sandwich structures. A similar phenomenon has been described in the research of Rim et al [20]. The smaller displacement and shorter contact time both verify the better impact damage resistance of the sandwich panels with SMA hybrid face-sheets.

The impact energy is dissipated by sandwich panels through elastic, plastic deformation and various damage modes, mainly including matrix cracks, delamination, fiber break and foam core crush during an impact event [17]. The energy absorbed by elastic deformation of the structure will be released during the rebound phase. Due to the low failure strain of the glass fibers and brittle nature of the polymer, the composite face-sheets experience almost no plastic deformation. Therefore, most of the impact energy is dissipated by various failures induced in the sandwich panels. As indicated in Table 3, there is a small difference on the absorbed energy between the panels investigated in this paper. As discussed above with Figure 4, Figure 5, Figure 6, Figure 7 and Figure 8, compared to the sandwich panel without SMA wires, there is less damage induced in the sandwich panels with hybrid composite face-sheets. Hence, a large amount of impact energy is dissipated by SMA wires due to their stress-induced martensitic phase transformation performance [36,37]. As shown in Figure 1, due to the high failure strain and tensile strength, SMA wires can experience a large plastic deformation. Also, because of the superelastic deformation performance, SMA wires can absorb a large amount of impact energy with the closed region in the stress-strain curve. Moreover, the SMA/polymer debonding can also contribute to the dissipation of the impact energy. Therefore, the impact damage resistance of the sandwich panels with SMA hybrid face-sheets is improved and less damage is induced compared to the conventional sandwich panel.

Based on the discussion above of the sandwich panels with one layer of SMA wires, modes IV and V, namely, the SMA wires are incorporated into the bottom face-sheet, having a better effect on the impact damage resistance of the structures, compared to modes II and III. The excellent mechanical performance of SMA wires can be fully exploited when embedded into the lower part of the structure, which has also been indicated in the SMA hybrid composite laminates subjected to low-velocity impact [29,34].

### 3.3. Impact Responses of Hybrid Sandwich Panels with Two Layers of SMA Wires

**Figure 10:**
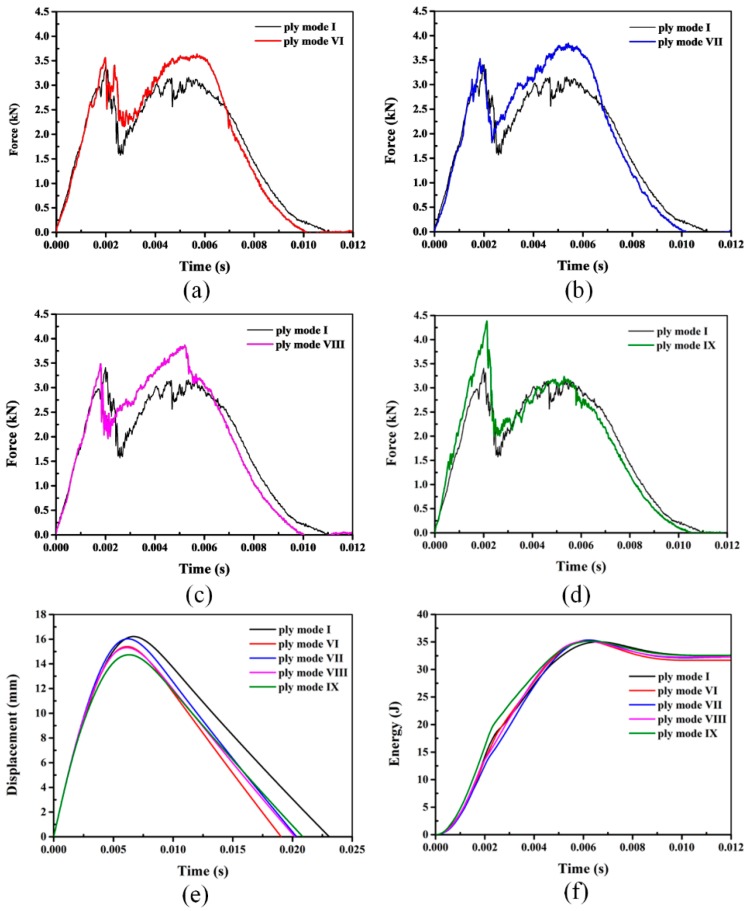
Typical impact responses of hybrid sandwich panels with two layers of SMA wires. (**a–d**) represent the force-time curves of mode I with modes VI, mode VII, mode VIII and mode IX, respectively; (**e**,**f**) show the displacement-time and energy-time histories, respectively.

The typical impact responses of hybrid sandwich panels with two layers of SMA wires is plotted in Figure 10. As shown for the hybrid sandwich panels with one layer of SMA wires in Figure 9, there are also two peak forces observed in the force-time curves of Figure 10. The first peak force still represents the load-bearing capability of the front face-sheet and the second indicates the indentation of the projectile into the core. The maximum contact forces of different sandwich panels are summarized in Table 4. It is clearly seen that the maximum forces of sandwich panels with hybrid face-sheets are larger than those of mode I. The maximum force of modes VI, VII, VIII and IX shows an improvement of 11.73%, 14.96%, 15.94% and 28.15%, respectively, relative to mode I. Compared to the sandwich panels with one layer of SMA wires, the maximum forces of sandwich panels with two layers of SMA wires are larger, which indicates a better load-bearing capability. The reasons for the better load-bearing capability of sandwich panels with two layers of SMA wires can be ascribed to the following two points. On the one hand, it is apparent that the volume fraction of SMA wires is increased. On the other hand, except for mode VI, at least one layer of SMA wires is incorporated into the bottom face-sheet. As indicated for hybrid sandwich panels with one layer of SMA wires, better impact damage resistance is acquired when SMA wires are embedded into the bottom face-sheet. Therefore, the hybrid face-sheets with two layers of SMA wires have a much better effect on the impact damage tolerance of the sandwich panels, and the best impact damage resistance of the structure is obtained when both layers of SMA wires are incorporated into the bottom face-sheet.

The typical displacement-time histories of hybrid sandwich panels with two layers of SMA wires are shown in Figure 10e. The maximum displacements of hybrid sandwich panels are much smaller than those of mode I. Table 4 clearly summarizes the maximum displacements of hybrid sandwich panels and the reference panel without SMA wires. Compared to mode I, the maximum displacements of modes VI, VII, VIII and IX decrease by 7.32%, 6.22%, 7.82% and 10.03% respectively. In general, it can be seen that the maximum displacements of hybrid sandwich panels with two layers of SMA wires are also smaller than the structures with one layer of SMA wires shown in Table 3. With a larger volume fraction of embedded SMA wires, the stiffness and strength of the structure are higher. Hence, the structure deformation is more reduced, and much better impact damage resistance is acquired. Similar to the sandwich panels with one layer of SMA wires, the time for the projectile to return the contact position during impact events with two layers of SMA wires is also shorter than that for the reference sandwich panel, which again verifies the improvement of the impact damage resistance due to the hybridization of SMA wires.

The absorbed energy of hybrid sandwich panels with two layers of SMA wires is also summarized in Table 4. As the results for panels with one layer of SMA wires, the absorbed energy of sandwich panels with two layers of SMA wires makes no large difference to the structure without SMA wires. As discussed in the previous section, the impact energy is mainly dissipated by different damages. The discussion in the previous section has indicated that the extent of the induced damage in the hybrid sandwich panels is much weaker than the panel without SMA wires. Compared to the reference sandwich panel, the delamination area in the face-sheets and the extent of the crushed foam core are greatly reduced. Especially, the delamination area and the crushed foam core depth of mode IX are the smallest, which indicates the best impact damage resistance can be obtained when the two layers of SMA wires are incorporated into the bottom face-sheets. The reason for the better impact resistance of hybrid sandwich panels is mainly attributed to the superelastic mechanical performance of SMA wires revealed in the previous section. Combining the discussions of the maximum force and displacement, it can be concluded that the optimal design for hybrid sandwich panels with two layers of SMA wires is mode IX, namely, both two layers of SMA wires are embedded in the bottom face-sheet.

## 4. Conclusions

New foam core sandwich panels with SMA hybrid face-sheets are designed for better impact damage resistance. The low-velocity impact tests on eight types of hybrid sandwich panels with one or two layers of SMA wires are carried out at the impact energy of 35 J. The following conclusions can be drawn:

(1) Compared to the sandwich panel without SMA wires, there is much less damage induced in the sandwich panels with SMA hybrid face-sheets. The damage area in the face-sheets and the extent of the crushed foam core are greatly reduced. Especially, when one layer of SMA wires is incorporated into the structure, the damage area shows a reduction of 13.74% in the front face-sheet and 85.63% in the bottom face-sheet for mode V; when two layers of SMA wires are applied, the damage area decreases by 37.93% in the front face-sheet and 75.69% in the bottom face-sheet.

(2) Compared with the structure without SMA wires, the load-bearing capability of the SMA hybrid sandwich panels is improved with higher maximum force. The maximum displacement of the SMA hybrid structures is smaller than the reference panel. For mode V, the maximum force increases by 10.26% and the maximum displacement decreases by 7.02% relative to the reference structure. For mode IX, the maximum force has an improvement of 28.15% and the maximum displacement shows a reduction of 10.03% compared to the reference panel.

(3) The impact damage resistance of sandwich panels with SMA hybrid face-sheets is improved due to the hybridization of SMA wires. As the volume fraction of SMA wires increases, the impact damage resistance of the structure is enhanced. For one layer of SMA wires applied in the structure, better impact damage resistance is acquired using the design of mode V in which the SMA wires are embedded into the lowest part of the structure. For two layers of SMA wires, the optimal design to improve the impact damage resistance of sandwich panels is mode IX, namely, both layers are incorporated into the bottom face-sheet.

## Figures and Tables

**Table 1:** The main mechanical characteristics of SMA wires [24].

Material	Modulus (GPa)	Tensile Strength (MPa)	Failure Strain (%)	Upper Plateau Stress (MPa)	Lower Plateau Stress (MPa)	Recoverable Strain (%)
NiTi	60.6	1522.7	12.4	530.8	170.8	7.9

**Table 2:** The specific locations of the SMA wires in the sandwich panels.

Category	Ply Mode	Stacking Sequence
Without SMA	Ply mode I	0°/0°/90°/90°/foam core/90°/90°/0°/0°
With SMA	Ply mode II	0°/SMA/0°/90°/90°/foam core/90°/90°/0°/0°
	Ply mode III	0°/0°/90°/SMA/90°/foam core/90°/90°/0°/0°
	Ply mode IV	0°/0°/90°/90°/foam core/90°/SMA/90°/0°/0°
	Ply mode V	0°/0°/90°/90°/foam core/90°/90°/0°/SMA/0°
	Ply mode VI	0°/SMA/0°/90°/SMA/90°/foam core/90°/90°/0°/0°
	Ply mode VII	0°/SMA/0°/90°/90°/foam core/90°/SMA/90°/0°/0°
	Ply mode VIII	0°/0°/90°/SMA/90°/foam core/90°/90°/0°/SMA/0°
	Ply mode IX	0°/0°/90°/90°/foam core/90°/SMA/90°/0°/SMA/0°

**Table 3:** Impact responses of the sandwich panels without or with one layer of SMA wires.

Mode	Maximum Force (kN)	Maximum Displacement (mm)	Energy (J)
I	3.41 ± 0.08	16.25 ± 0.09	32.65 ± 0.13
II	3.58 ± 0.11	15.31 ± 0.12	32.81 ± 0.16
III	3.78 ± 0.12	15.66 ± 0.16	32.87 ± 0.17
IV	3.74 ± 0.08	15.15 ± 0.14	31.52 ± 0.13
V	3.76 ± 0.09	15.11 ± 0.12	31.31 ± 0.15

**Table 4:** Impact responses of the sandwich panels without or with two layers of SMA wires.

Mode	Maximum Force (kN)	Maximum Displacement (mm)	Energy (J)
I	3.41 ± 0.08	16.25 ± 0.09	32.65 ± 0.13
VI	3.81 ± 0.13	15.06 ± 0.22	31.67 ± 0.21
VII	3.92 ± 0.17	15.24 ± 0.39	32.15 ± 0.27
VIII	3.95 ± 0.16	14.98 ± 0.25	32.17 ± 0.23
IX	4.37 ± 0.12	14.62 ± 0.21	32.52 ± 0.19
